# CG14906 (mettl4) mediates m^6^A methylation of U2 snRNA in *Drosophila*

**DOI:** 10.1038/s41421-020-0178-7

**Published:** 2020-06-30

**Authors:** Lei Gu, Longfei Wang, Hao Chen, Jiaxu Hong, Zhangfei Shen, Abhinav Dhall, Taotao Lao, Chaozhong Liu, Zheng Wang, Yifan Xu, Hong-Wen Tang, Damayanti Chakraborty, Jiekai Chen, Zhihua Liu, Dragana Rogulja, Norbert Perrimon, Hao Wu, Yang Shi

**Affiliations:** 1 Department of Medicine, Division of Newborn Medicine and Epigenetics Programe, Boston Children’s Hospital, Boston, MA 02115 USA; 2 Department of Cell Biology, Harvard Medical School, Boston, MA 02115 USA; 3 Department of Biological Chemistry and Molecular Pharmacology, Harvard Medical School, Boston, MA 02115 USA; 4 Program in Cellular and Molecular Medicine, Boston Children’s Hospital, Boston, MA 02115 USA; 5 Department of Ophthalmology and Vision Science, Shanghai Eye, Ear, Nose and Throat Hospital, Fudan University, 200031 Shanghai, China; 6 Department of Ophthalmology, The Affiliated Hospital of Guizhou Medical University, Guiyang, Guizhou 550004 China; 7 Division of Rheumatology, Allergy and Immunology, Center for Immunology and Inflammatory Diseases, Massachusetts General Hospital, Charlestown, MA 02129 USA; 8 Department of Genetics, Harvard Medical School, 77 Avenue Louis Pasteur, Boston, MA 02115 USA; 9 CAS Key Laboratory of Regenerative Biology, Joint School of Life Sciences, Guangzhou Institutes of Biomedicine and Health, Chinese Academy of Sciences, Guangzhou Medical University, Guangzhou, Guangdong 510530 China; 10 Department of Neurobiology, Harvard Medical School, Boston, MA 02115 USA; 11 Howard Hughes Medical Institute, 77 Avenue Louis Pasteur, Boston, MA 02115 USA

**Keywords:** DNA methylation, Transcriptional regulatory elements

Dear Editor,

While the eukaryotic candidate m^6^A methyltransferases belong to multiple distinct methylase lineages, the most widespread group belongs to the MT-A70 family exemplified by the yeast messenger RNA (mRNA) adenine methylase complex Ime4/Kar4. At the structural level, all of these enzymes are characterized by a 7-β-strand methyltransferase domain at their C terminus, fused to a predicted α-helical domain at their N terminus and require *S*-adenosyl-l-methionine (SAM) as a methyl donor. The catalytic motif, [DSH]PP[YFW], present in many members of this family, has shown to be critical for METTL3/METTL4-mediated mRNA m^6^A methylation^1^. The high degree of amino acid sequence conservation among the predicted N6-methyladenosine methyltransferases motivates further explorations into their potential functional conservation. METTL4 is a member of the MT-A70-like protein family, which is conserved during evolution (Fig. 1a)^2^. Previous studies suggested that METTL4 regulates DNA 6mA in vivo and therefore is a candidate DNA 6mA methyltransferase^3–5^. However, the enzymatic activity of METTL4 in vitro has not been demonstrated.

**Fig. 1:**
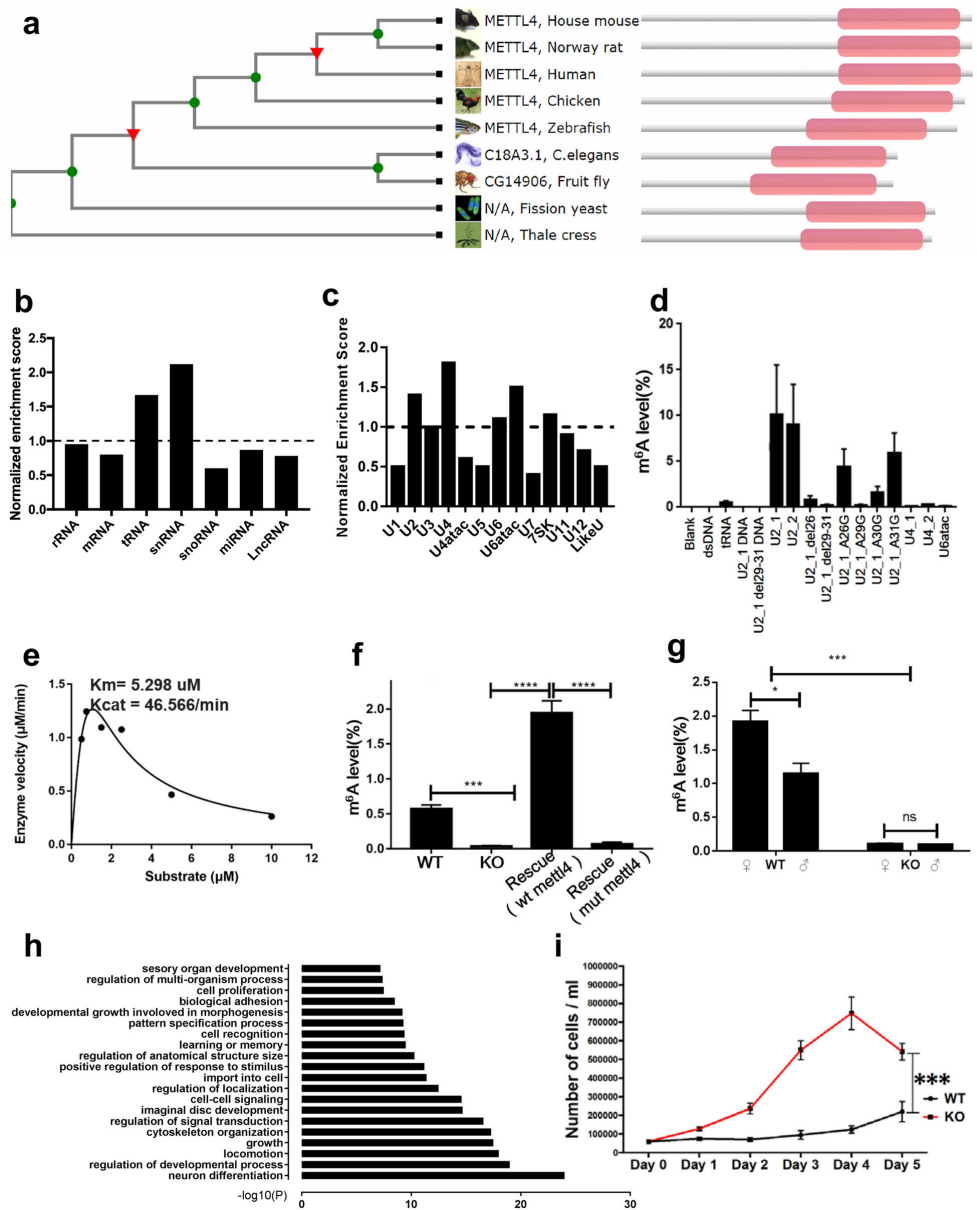
CG14906 (mettl4) methylates U2 snRNA in *Drosophila melanogaster*. **a** Cladogram of mettl4 in model organisms based on their sequence similarity, the pink rectangle indicates the MT-A70 domain. **b** Enrichment analysis of eCLIP-seq data for different RNA types. tRNA and snRNA are enriched among all RNA types in general and snRNA is the top enriched RNA molecules targeted by mettl4 in vivo. **c** Enrichment analysis of eCLIP-seq data for subgroups of snRNA. U2, U4, and U6atac are the top enriched subgroups. **d** In vitro enzymatic activity is measured by LS-MS/MS using substrates, including U2, U4, U6atac, and U2, with different point mutations and deletions, and tRNA and DNA with U2 sequences. Results show that U2 is the best substrate for fly mettl4 and that adenosine at position 29 in U2 is methylated by mettl4. **e** Michaelis–Menten kinetics of recombinant *mettl4* was determined using U2 probes as substrate by LC-MS/MS analysis. **f** U2 m^6^A analysis in WT, KO, and rescued (wt: wild-type *mettl4*; mut: catalytic dead mutant *mettl4*, DPPW→NPPW) cells by LC-MS/MS. **g** In vivo U2 m^6^A analysis by LC-MS/MS of WT and KO flies. Error bars indicate mean ± SD (*n* = 3). **h** Genes with differential alternative splicing were used for the GO analysis. The top 20 enriched biological processes are shown in the bar plot. **i** Growth curves of WT and *mettl4* KO cells in a course of 5 days. Error bars indicate mean ± SD (*n* = 7). Permutation test was used to determine the significant level of the difference between two groups of growth curves. Statistical significance is determined as: n.s., *P* > 0.05; *P < 0.05; ***P < 0.001; ****P < 0.0001.

To identify the substrate(s) for METTL4, we purified His-tagged, wild-type (WT) as well as a catalytic mutant (DPPW mutated to NPPW) (Supplementary Fig. S1) of *Drosophila melanogaster* mettl4 from *Escherichia coli* strain BL21 (DE3). In order to unbiasedly identify potential substrates of mettl4, we performed in vitro enzymatic assays using various substrates, including both DNA and RNA with and without secondary structures. We used deuterated *S*-adenosyl methionine (SAM-d3) in the in vitro enzymatic assays in order to identify m^6^A mediated by mettl4. Although we detected a weak enzymatic activity on DNA substrates composed of previously published sequence motifs, mettl4 appears to prefer RNA substrates potentially with secondary structures (Supplementary Fig. S2). We next performed enhanced crosslinking and immunoprecipitation (IP) followed by high-throughput sequencing (eCLIP-seq), which was originally developed to map binding sites of RNA-binding proteins on their target RNAs^6^, to identify the RNA type that is targeted by mettl4 in vivo. Since there are no commercial antibodies available for fly mettl4, we generated a *Drosophila* Kc cell line with a FLAG-tagged mettl4 for the eCLIP-seq experiment^7^. In total, we generated two biological replicates for IP samples, and their respective input samples, together with one IP-control and input-control sample for the quality control and enrichment analysis^8^. The two replicates showed a strong correlation with a Spearman’s correlation coefficient of 0.97, indicating great consistency between the replicates (Supplementary Fig. S3). Thus, we merged the two replicates to increase the sequencing depth and power for downstream analyses, which showed that mettl4 captured RNA molecules, mostly transfer RNA (tRNA) and small nuclear RNA (snRNA), including U2, U4, and U6atac (Fig. 1b, c).

We next investigated whether the RNAs identified by the eCLIP experiments are indeed substrates of mettl4 by carrying out in vitro enzymatic assays. We synthesized oligonucleotides containing tRNA and snRNA sequences and various controls, including DNAs with the same sequences (Supplementary Table S1). The in vitro enzymatic activity of mettl4 on each candidate substrate and control sequences was measured by liquid chromatography with tandem mass spectrometry (LC-MS/MS). These in vitro experiments led to the identification of U2 as the best substrate among all the snRNA subtypes (Fig. 1d). Next, we wished to identify the adenosine residues in U2 that are methylated by mettl4. Previous studies documented that the adenosine at the 30th position of U2 is frequently methylated in vertebrate U2 snRNA^9^, with a sequence motif of AA-G as opposed to _29_AAAG_32_ in fly. To identify which adenosine within the motif is essential for the enzymatic activity in fly, we generated point mutations and deletions of adenosine within and close to this motif and measured the enzymatic activity of mettl4 on these substrates. We found that when the 29th position adenosine is mutated or deleted, no m^6^A methylation was detected by LC-MS/MS, whereas other point mutations or deletions (26th and 31st positions) did not affect substrate methylation or only decreased methylation partially (i.e., the 30th position). These results indicate that adenosine at position 29 is the adenosine in U2 that is methylated by mettl4 in fly (Fig. 1d). In order to better characterize the enzymology of mettl4, we next investigated the kinetics of mettl4 and determined that mettl4 was able to methylate U2 with a Michaelis–Menten constant (*K*_m_) of 5.298 μM and a catalytic rate constant (*k*_cat_) of 46.566 min^−1^ (Fig. 1e). In addition, the enzyme is inhibited by the substrate at higher concentrations (Fig. 1e).

Next, we investigated whether mettl4 catalyzes U2 m^6^A in vivo. To accomplish this goal, we generated *mettl4* knockout (KO) and rescue cell lines (rescued by either WT or catalytic mutant of mettl4) (Supplementary Figs. S4 and S5). Indeed, the U2 m^6^A level is decreased dramatically in the *mettl4* KO cells and restored in the wt, but not in the catalytic compriomised, *mettl4* rescued cells (Fig. 1f; Supplementary Figs. S7 and S8a). Furthermore, the same reduction of U2 m^6^A level was also seen in KO flies (Fig. 1g; Supplementary Figs. S6 and S8b). The low DNA 6mA levels between WT and KO fly cells for both nuclear and mitochondrial DNA showed no significant differences (Supplementary Fig. S9). These findings suggest that it is mettl4 that mediates U2 methylation in vivo. Interestingly, the U2 m^6^A level in WT female flies is significantly higher than that in males, suggesting that mettl4 might play sex-specific roles (Fig. 1g; Supplementary Fig. S8b), which will be interesting to investigate in the future. Given U2 snRNA is involved in pre-mRNA splicing^10^, we performed RNA-seq using both WT and *mettl4* KO *Drosophila* Kc cell lines to determine if RNA splicing is affected as a result of mettl4 loss. In total, we identified 2366 transcripts with differential alternative splicing events, which cover 1771 genes. Gene Ontology Enrichment analysis suggests that *mettl4* affects a broad set of biological processes, including differentiation, development, growth, and response to stimulus (Fig. 1h). We next investigated whether there are any significant phenotypic differences between WT and KO cells, given the broad changes in the whole transcriptome caused by *mettl4* KO. Indeed, we observed a significant proliferation difference between WT and *mettl4* KO cells (Fig. 1i; Supplementary Fig. S10). In addition, we analyzed independently generated *mettl4* knockdown (KD) cell lines by RNA interference (RNAi), and both KD cell lines displayed enhanced growth/proliferation than control cells (Supplementary Fig. S11), indicating loss of mettl4 is associated with enhanced cell proliferation. Although both KO and KD cell lines show similar proliferation pattern, genetic rescue experiments are needed to definitively rule out potential off-target effects.

Since U2 is an essential component of the major spliceosomal complex, which plays an important role in pre-RNA splicing, loss of *mettl4* might have broad impacts through altered RNA splicing. However, whether the altered RNA splicing events are regulated by mettl4 through methylation of U2 snRNA or other yet-to-be-identified substrates, or whether mettl4 regulates splicing in an enzymatic activity-independent manner, remain to be determined in the future. In addition, we did not observe any significant difference during development of *mettl4* KO flies, although we observed altered proliferation of the Kc cell line lacking *mettl4*. The reason for the discrepancy between cell line and whole fly studies is unclear at this time. Gene expression at the organismal level is regulated in a spatiotemporal manner and by both genetic and environmental factors^11,12^. This complexity may explain why we did not observe overt developmental phenotypes of the *mettl4* KO fly.

At the same time, we also identified METTL4 as a novel methyltransferase for U2 snRNA in human. Human METTL4 catalyzes Am to m^6^Am, whereas fly mettl4 catalyzes A to m^6^A. Although the only difference between m^6^Am and m^6^A is the 2′-O-methyl group on the sugar, we demonstrated that human METTL4 cannot convert A to m^6^A. Future structural studies will provide insight into how these two highly related enzymes come to possess different substrate requirements for m^6^A methylation of U2 snRNA. Furthermore, since the U2 RNA undergoes different modifications (m^6^A vs m^6^Am), it is possible that they could have distinct biological functions and significances. They may affect the structure and function of the U2 RNA or even the spliceosome differently, and require different readers and erasers, as well as a set of Am writers/readers/erasers. Indeed, while fly cells lacking *mettl4* show an enhanced proliferation rate, human 293T cells do not. Consistently, pathway analysis shows that cell proliferation genes are affected in response to *mettl4* loss only in fly, but not in human cells (293T). Together, these findings raise many intriguing questions, including the origin of the substrate preference, the structural mechanism that contributes to the recognition of the 2′-O-methyl group on Am, and the biological implications of the mechanistic evolution of METTL4.

In summary, we demonstrated that mettl4 catalyzes U2 m^6^A in fly both in vitro and in vivo. Furthermore, whole transcriptome profiling revealed that loss of *mettl4* broadly impacts various biological pathways. Lastly, we were able to observe a significant difference in cell proliferation between *mettl4* normal and deficient fly cells. Our work answered a long-standing question regarding the enzymatic activity of mettl4, and thus paved the way for further investigation of mettl4 functions in different biological settings.

## Supplementary information

Supplementary Information

## Supplementary information

**Supplementary Information** accompanies the paper at (10.1038/s41421-020-0178-7).

## References

[1] Iyer LM, Zhang D, Aravind L (2016). Adenine methylation in eukaryotes: apprehending the complex evolutionary history and functional potential of an epigenetic modification. BioEssays.

[2] Iyer LM, Abhiman S, Aravind L (2011). Natural history of eukaryotic DNA methylation systems. Prog. Mol. Biol. Transl. Sci..

[3] Kweon SM (2019). An adversarial DNA N(6)-methyladenine-sensor network preserves polycomb silencing. Mol. Cell.

[4] Ma C (2019). N6-methyldeoxyadenine is a transgenerational epigenetic signal for mitochondrial stress adaptation. Nat. Cell Biol..

[5] Greer EL (2015). DNA methylation on N6-adenine in *C. elegans*. Cell.

[6] Van Nostrand EL (2016). Robust transcriptome-wide discovery of RNA-binding protein binding sites with enhanced CLIP (eCLIP). Nat. Methods.

[7] Van Nostrand EL (2017). CRISPR/Cas9-mediated integration enables TAG-eCLIP of endogenously tagged RNA binding proteins. Methods.

[8] Van Nostrand EL, Huelga SC, Yeo GW (2016). Experimental and computational considerations in the study of RNA-binding protein–RNA interactions. Adv. Exp. Med. Biol..

[9] Karijolich J, Yu YT (2010). Spliceosomal snRNA modifications and their function. RNA Biol..

[10] Lee Y, Rio DC (2015). Mechanisms and regulation of alternative pre-mRNA splicing. Annu. Rev. Biochem..

[11] McGuire SE, Mao Z, Davis RL (2004). Spatiotemporal gene expression targeting with the TARGET and gene-switch systems in *Drosophila*. Sci. STKE.

[12] Greene CS (2015). Understanding multicellular function and disease with human tissue-specific networks. Nat. Genet..

