# Supplementary material for: CG14906 (mettl4) mediates m6A methylation of U2 snRNA in Drosophila
Source: Cell Discov. 2020 Jun 30;6:44. doi: 10.1038/s41421-020-0178-7 (PMC7324582; doi:10.1038/s41421-020-0178-7)
Supplement: Supplementary file 1 — Supplementary Information [file 41421_2020_178_MOESM1_ESM.pdf]

Supplementary Fig. S1 SDS-PAGE of purified protein encoded by CG14906 (*mettl4*)

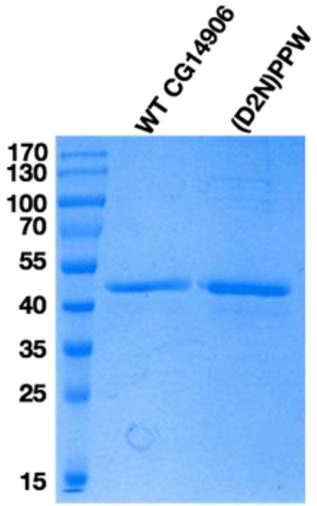

Supplementary Fig. S1 SDS-PAGE of recombinant proteins encoded by CG14906 (*mettl4*). His-tagged recombinant *Drosophila* melanogaster *mettl4* proteins (wildtype and a catalytic mutant with a point mutation in the DPPW motif: DPPW to NPPW) were purified from the *E.coli* strain BL21 (DE3).

Supplementary Fig. S2 *in vitro* enzymatic activity of Drosophila *mettl4* on DNA and RNA substrates with different sequences

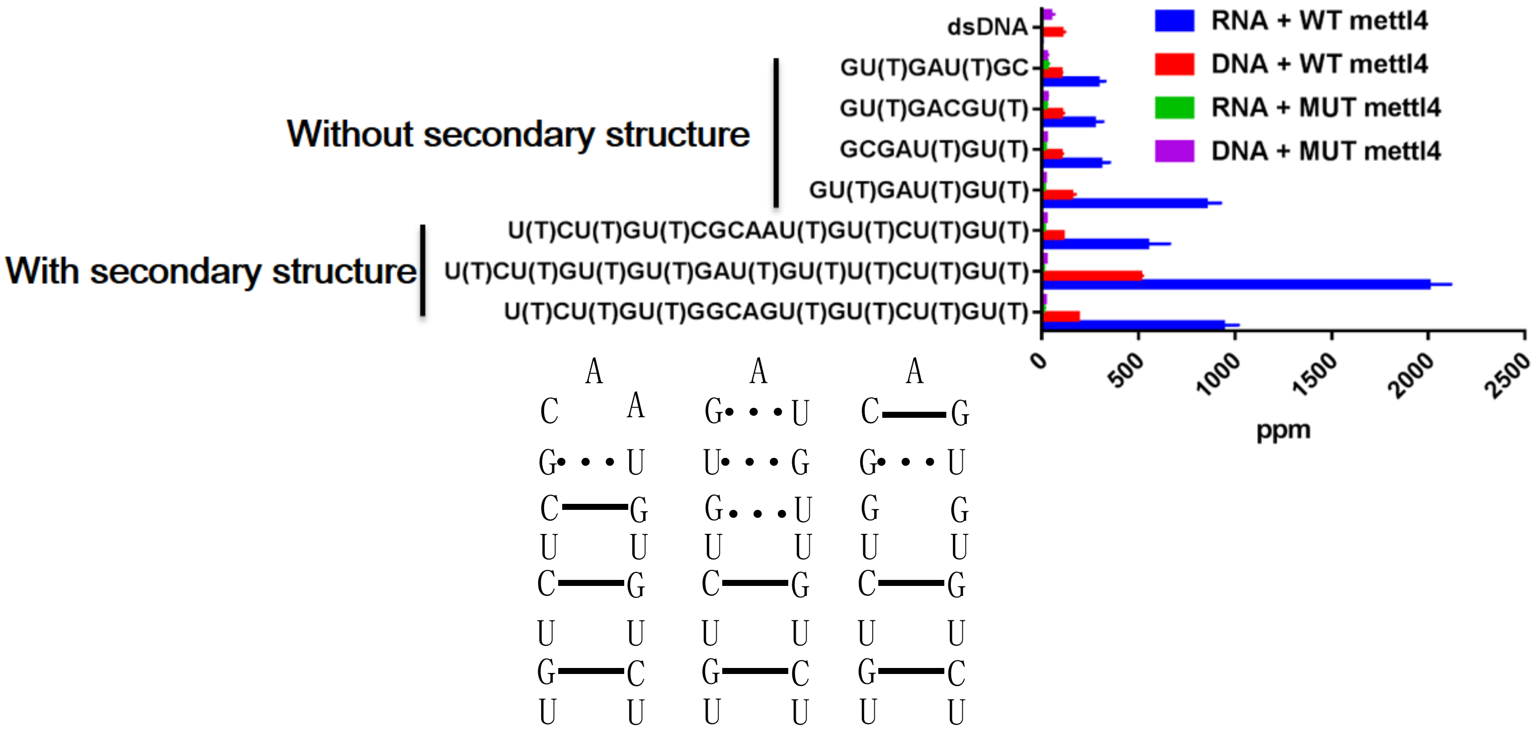

Supplementary Fig. S2 *In vitro* enzymatic activity of Drosophila *mettl4* on DNA and RNA substrates with different sequences. Both DNA and RNA substrates, upon incubation with the recombinant *mettl4*, were measured for the m6A level by LC-MS/MS. A low level of m6A was observed in the DNA substrates and a relatively high level of m6A was observed in structured RNA substrates. Error bars indicate mean ± s.d. (n=3).

Supplementary Fig. S3 Correlation between biological replicates for the eCLIP-seq samples

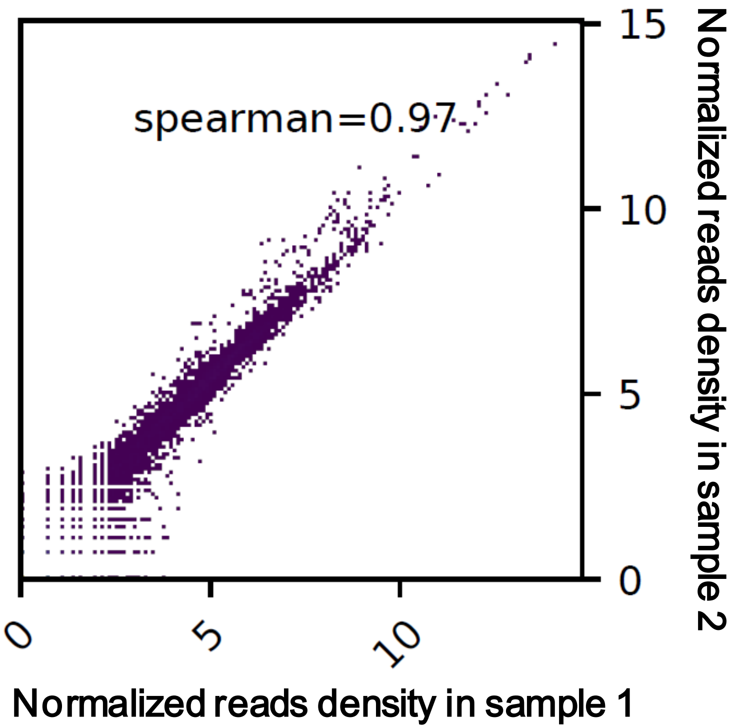

Supplementary Fig. S3 Correlation between biological replicates of the eCLIP-seq samples. Reads aligned to fly reference genome (dm6) were normalized in reads per million and the reads density for each 1-kb window was used for the Spearman correlation coefficient calculation.

Supplementary Fig. S4 Generation of knockout Kc cell line

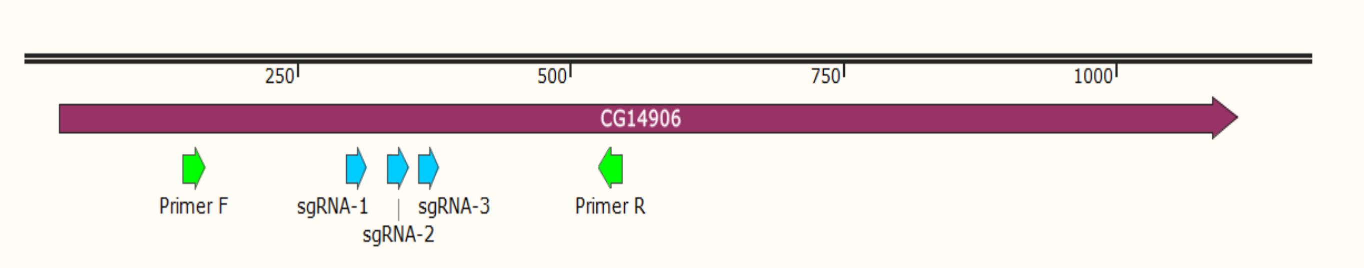

PCR product: 402bp

Small indels produced by single guide RNA caused frame shift mutations.

sgRNA-1: Editing rate: 90% sgRNA-2: Editing rate: 90% sgRNA-3: Editing rate: 90%

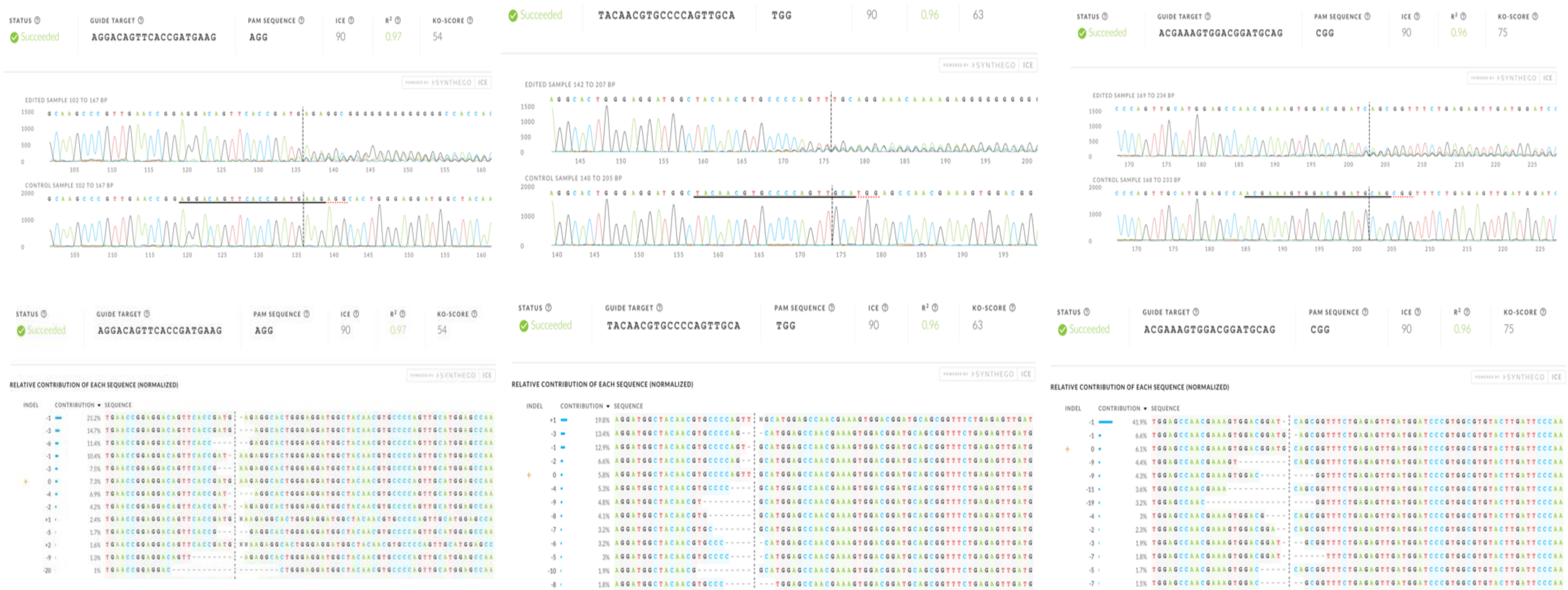

Supplementary Fig. S4 Generation of a knockout Kc cell line. *mettl4* in fly Kc cell line was knocked out by CRISPR-Cas9. Small indels produced by a single guide RNA caused frame shift mutations and the editing efficiency was over 90% based on the ICE CRISPR Analysis Tool.

Supplementary Fig. S5 Rescue of m6A level of U2 snRNA by overexpressing *mettl4* in the *mettl4* KO cells

Pentry- PAWF Gateway system:

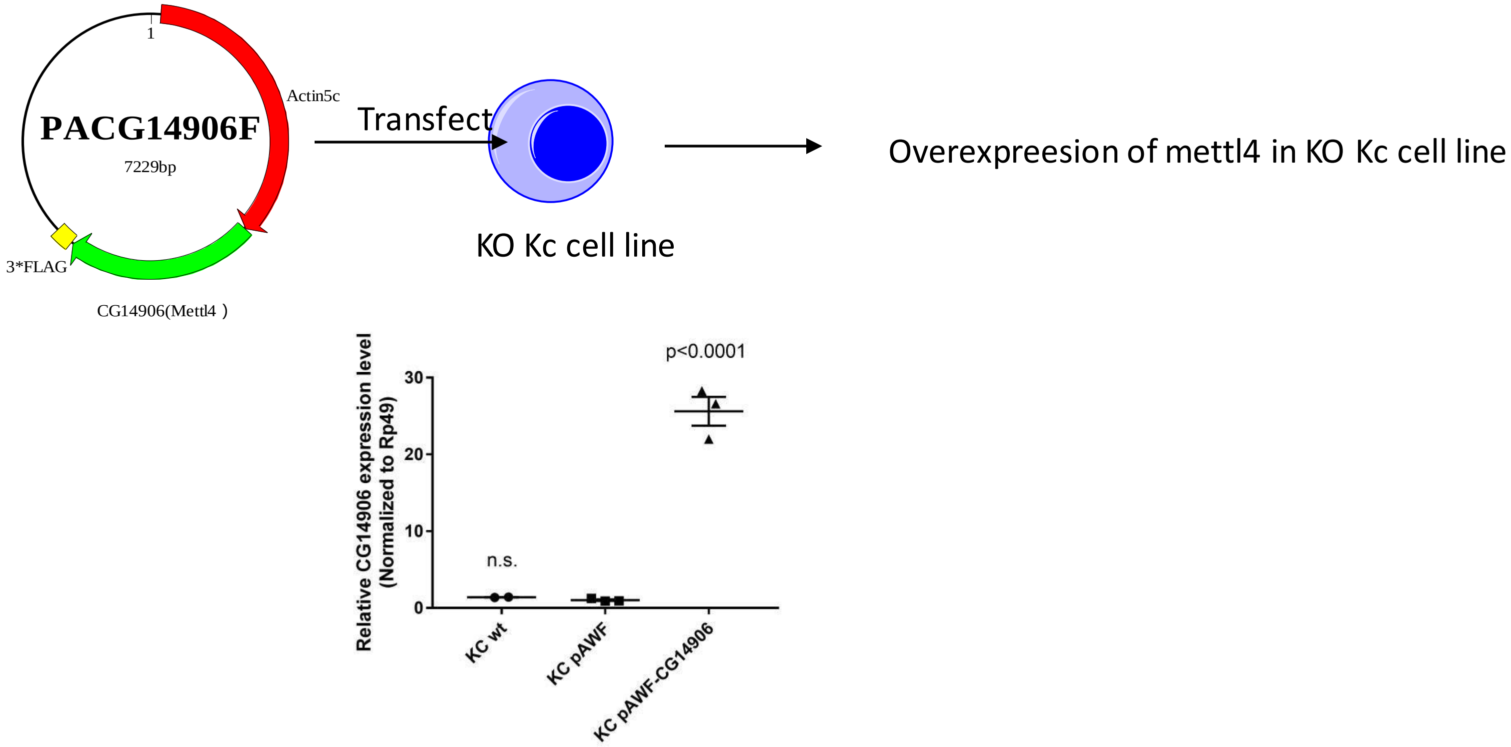

Values are shown as mean±SEM. Results are normalized to KC pAWF.

Supplementary Fig. S5 Rescue of m6A level of U2 snRNA by overexpressing *mettl4* in the *mettl4* KO cells. The Pentry-PAWF Gateway system was used to over-express *mettl4* in the fly Kc KO cells. The expression level of *mettl4* was measured by qPCR and normalized to Kc pAWF.

Supplementary Fig. S6 Generation of *mettl4* knockout fly

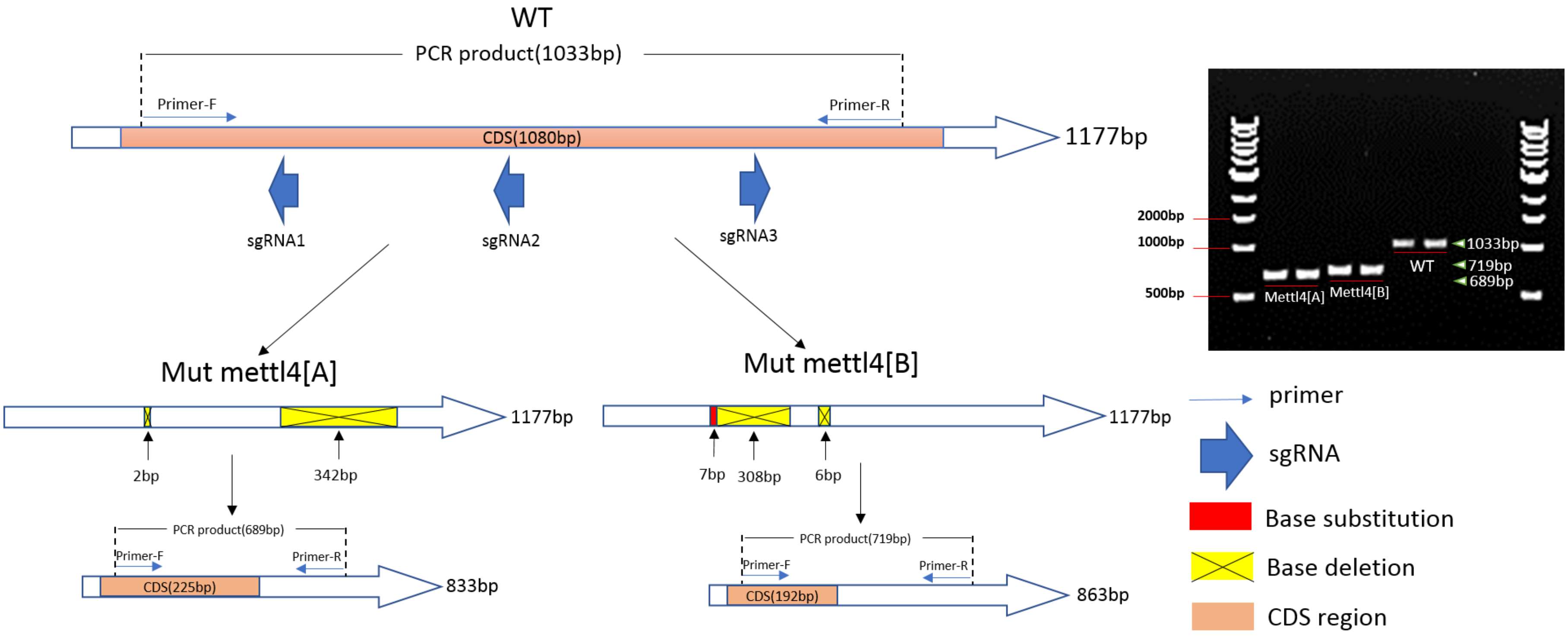

Supplementary Fig. S6 Generation of *mettl4* knockout fly. *mettl4* knockout flies were generated using the CRISPR/Cas9 system and verified by PCR.

Supplementary Fig. S7 MS spectra of U2 in *mettl4* WT, KO and the rescued cells

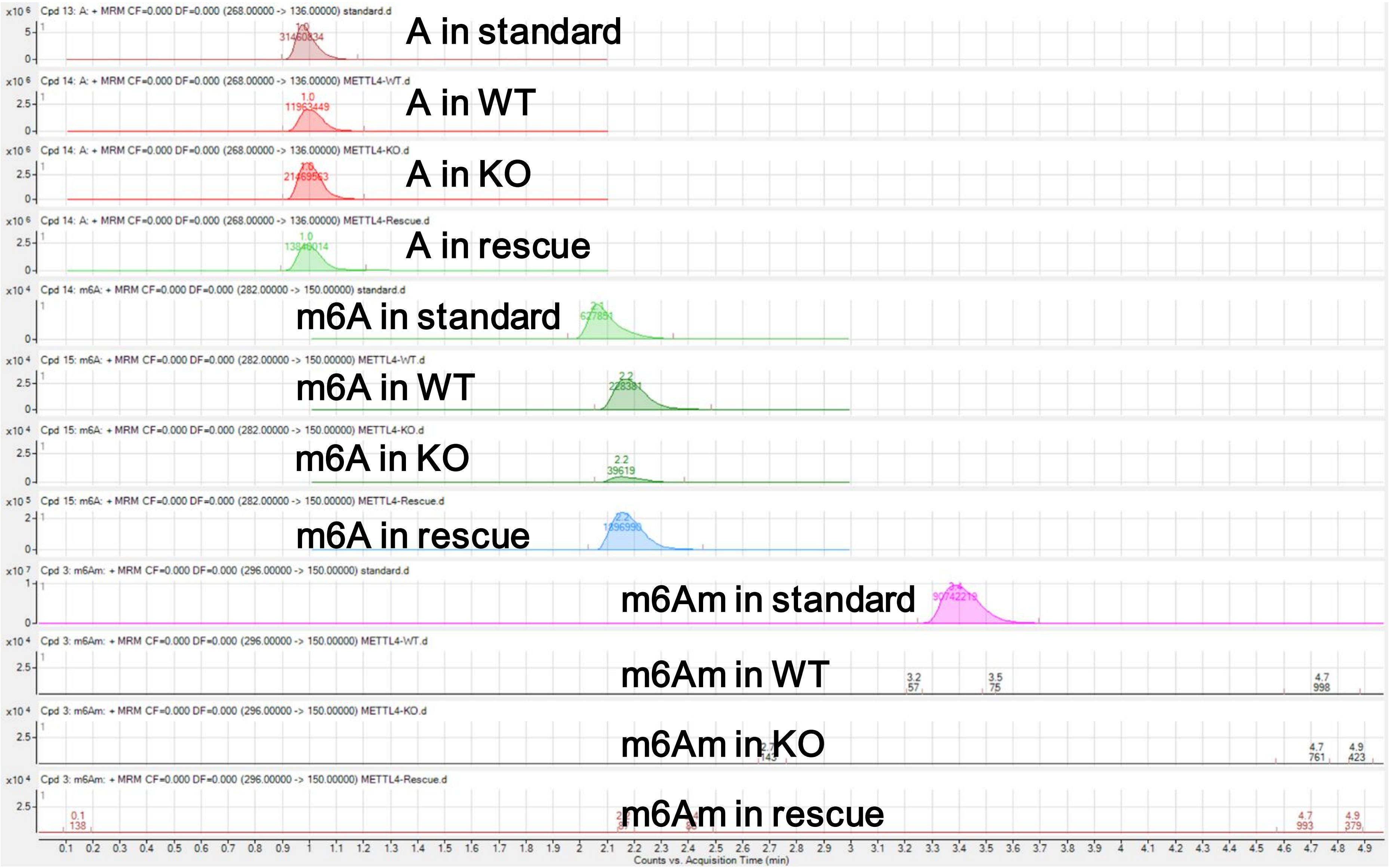

Supplementary Fig. S7 MS spectra of U2 in *mettl4* WT, KO and the rescued cells. A, m6A and m6Am levels were measured by LC-MS/MS and compared among WT, KO and rescued cells. Standard peaks indicate the retention time for each modification.

Supplementary Fig. S8 LC-MS/MS results for other independent KO cell lines and flies

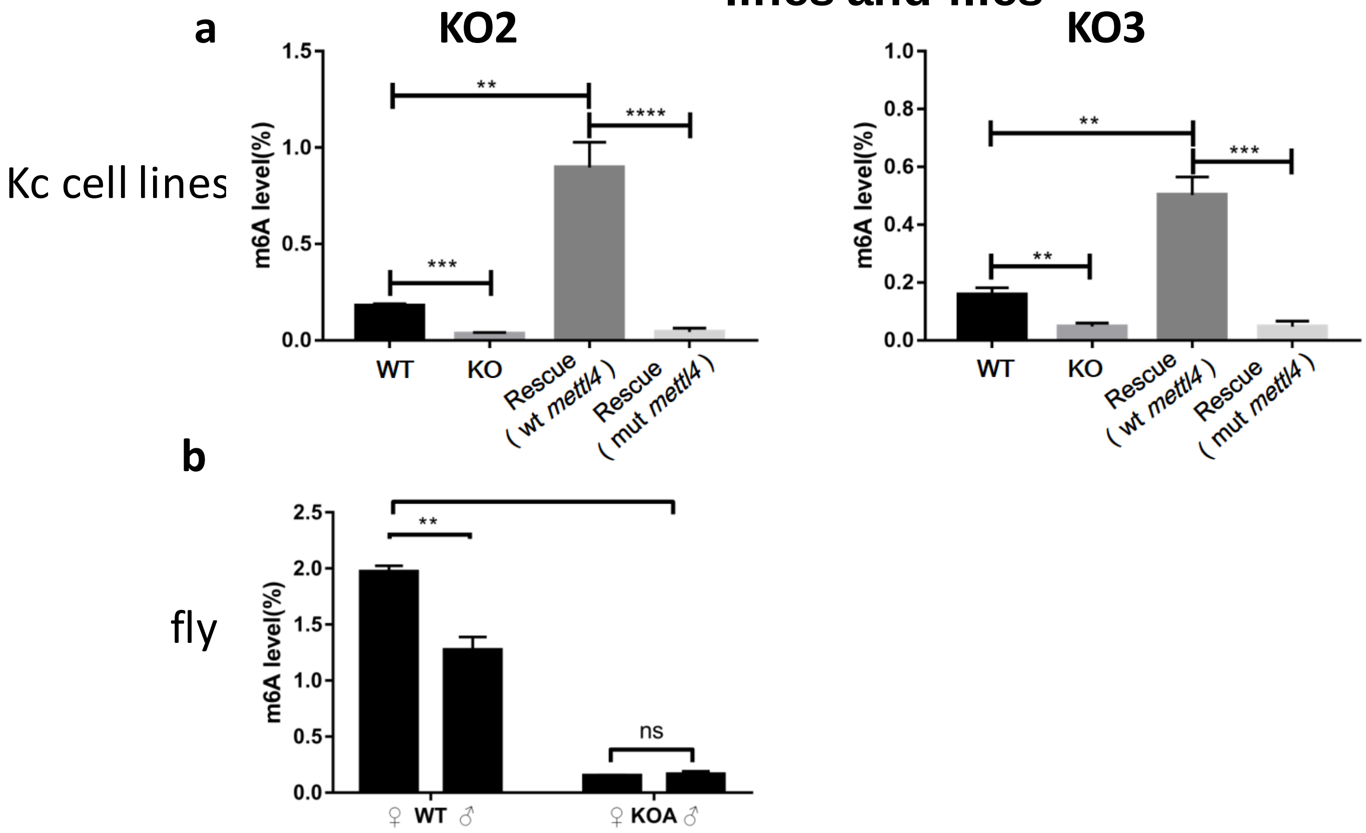

Supplementary Fig. S8 LC-MS/MS results for other independent KO cell lines and flies. **a.** m6A levels for the other two independent KO cell lines and **b.** one KO fly line were measured by LC-MS/MS. Error bars indicate mean ± s.d. (n=3). Statistical significance is determined as: ns = p > 0.05; * = p < 0.05; ** = p < 0.01; *** = p < 0.001; **** = p < 0.0001.

Supplementary Fig. S9 LC-MS/MS results for 6mA levels on nuclear and mitochondrial DNA from WT and KO fly cells

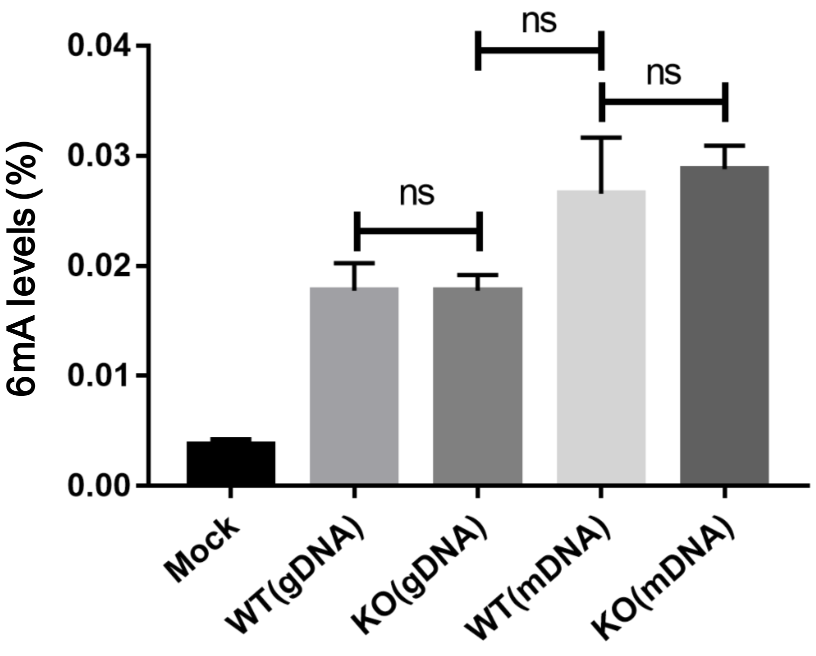

Supplementary Fig. S9 LC-MS/MS results for 6mA on nuclear and mitochondrial DNA from WT and KO fly cells. 6mA levels for both nuclear and mitochondrial DNA from WT and KO fly cells were measured by LC-MS/MS. Error bars indicate mean ± s.d. (n=3). Statistical significance is determined as: ns = $p > 0.05$.

Supplementary Fig. S10 cell proliferation pattern for other independent KO cell lines

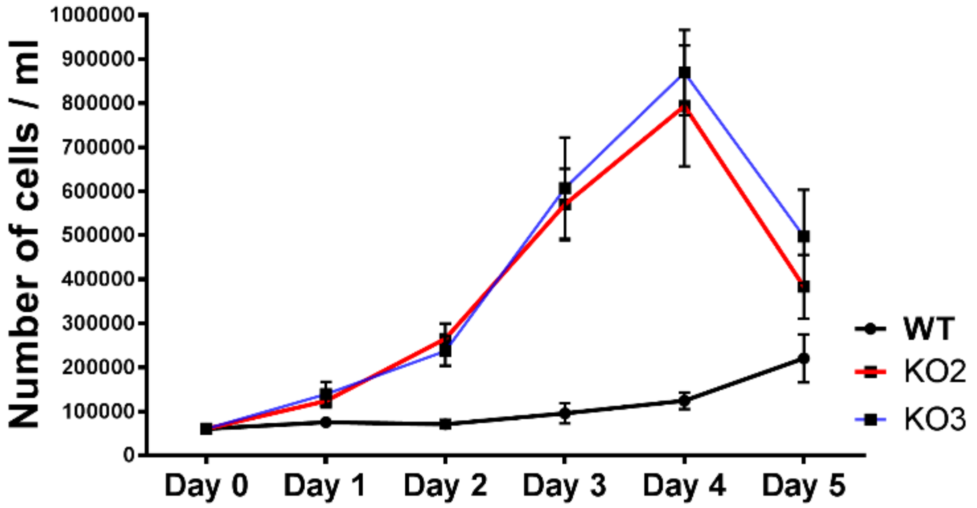

Supplementary Fig. S10 Cell proliferation pattern for other independent KO cell lines. Growth curves of *mettl4* WT and other two independent KO cells in a course of 5 days. Error bars indicate mean ± s.d. (n=7).

Supplementary Fig. S11 cell proliferation pattern for knock-down cell lines by RNAi

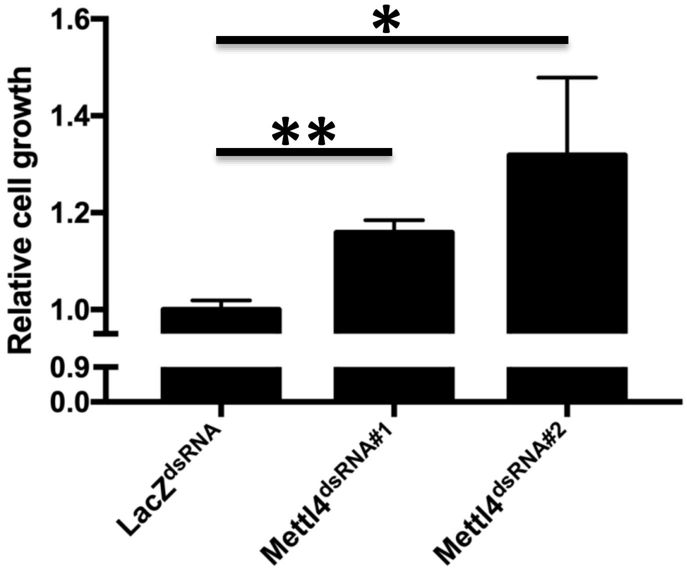

Supplementary Fig. S11 Cell proliferation pattern for knock-down cell lines by RNAi. Relative cell growth of Kc cells treated with dsRNAs against LacZ (control) and *mettl4* for 72hrs. Cells treated with *mettl4* ^{dsRNA} were normalized to control cells. t test was performed to identify significant differences between samples; Error bars indicate mean $\pm$ s.d. (n=3). Statistical significance is determined as: ns = $p > 0.05$; * = $p < 0.05$; ** = $p < 0.01$.

Supplementary Table S1 substrates' sequences for in vitro enzymatic activity assay

dsDNA: genomic DNA from cell line (GM12878)

tRNA: commercially available yeast tRNA extract

U2_1_DNA:
ATCGCTTCTCGGCCTTATGGCTAAGATCAAAGTGTAGTATCTGTTCTTATCAGCTTAACAT
CTGATAGTTCCTCCATTGGAGGACAACAAATGTTAAACTGATTTTTGGAATCAGACGGAG
TGCTAGGGGCTTGCTCCACCTCTGTCACGGGTTGGCCCGGTATTGCAGTACCGCCGGG
ATTTCGGCCCAAC

U2_1:
AUCGCUUCUCGGCCUUAUGGCUAAGAUCAAAGUGUAGUAUCUGUUCUUAUCAGCUUA
ACAUCUGAUAGUUCCUCCAUUGGAGGACAACAAAUGUUAAACUGAUUUUUGGAAUCAG
ACGGAGUGCUAGGGGCUUGCUCCACCUCUGUCACGGGUUGGCCCGGUAUUGCAGUA
CCGCCGGGAUUUCGGCCCAAC

U2_2:
AUCGCUUCUCGGCCUUAUGGCUAAGAUCAAAGUGUAGUAUCUGUUCUUAUCAGCUAA
CAUCUGAUAGUUCCUCCAUUGGAGGACAACAAAUGUUAAACUGAUUUUUGGAAUCAGA
CGGAGUGCUAGGGGCUUGCUCCACCUCUGUCACGGGUUGGCCCGGUAUUGCAGUAC
CGCCGGGAUUUCGGCCCAAC

U4_1:
AUCUUUGCGCAGUGGCAAUACCGUAACCAAUGAAGUCCUCCUGAGGUGCGGUUAUUG
CUAGUUGAAAACUUUAACCAAUACCCCGCCAUGGGGACGUGAAAUACCGUCCACUACG
GCAAUUUUUGGAAGCCCGAGAGGGCUAA

U4_2:
AUCUUUGCGCAGAGGCGAUAUCGUAACCAAUGAAGUUCUACUGAGGUGCGAUUAUUG
CUAGUUGAAAACUUUAACCAAUACCCCGCCAUGGGGACGUGAAAUACCGUCCACUACG
GCAAUUUUUGGAAGCCCGAGAGGGCCA

U6aUac:
GUGUUGUUUGGAAGGAGAGCAAGUUAGCACUCCCCUAGACAAGGAUGGAACACAUAA
ACGGUCGGCUAGGCACAGACAAAAGCCGUCCACAAAUUUU

Supplementary Information

Materials and Methods

Protein purification

Cells were harvested and sonicated in lysis buffer (25mM Tris-HCl [pH 8.0], 1M NaCl, 5% glycerol, 2mM dithiothreitol (DTT) and supplemented with protease inhibitors). MBP-tagged METTL4 was purified by affinity for maltose binding protein and the tag was removed by digestion with Tobacco Etch Virus (TEV) protease. Target proteins were further purified by HiTrap Heparin HP columns chromatography, gel filtration chromatography and then was analyzed by SDS-PAGE [1]. The final METTL4 in buffer containing 25 mM Tris (pH 8.0), 150 mM NaCl and 2mM DTT.

Overexpression of Target Proteins

For proteins expressed in E. coli, specified constructs of Drosolhila METTL4 was subcloned into pET28a vector and transformed into Rosetta (DE3) bacterial cells (Novagen). Cells were incubated with agitation at 37℃ until OD600 0.5-0.6, and then cells were induced by adding IPTG to 0.2 mM final concentration. Target proteins were further expressed at 20℃ overnight [2].

U2 snRNA purification

Total RNA was extracted from frozen flies with TRIzol reagent according to Invitrogen Life Technologies manual. Streptavidin-conjugated M-280 magnetic Dynabeads (Invitrogen) were used for specific U2 snRNA Isolation. RNase-free beads were washed once with buffer A (10 mM Tris-HCl, pH 7.5, 2 mM EDTA, 2M NaCl), and resuspended in buffer A. Subsequently, biotinylated oligonucleotides were mixed with Dynabeads in buffer A and incubated at room temperature for 30 min with gentle mixing. After the incubation, the oligonucleotide-coated Dynabeads were then washed for four times in buffer B (5 mMTris-HCl, pH 7.5, 1mM EDTA, 1MNaCl) and equilibrated in 6 x SSC solution (1 x SSC is 0.15M NaCl plus 0.015 M sodium citrate, pH 7.0). The oligonucleotide-coated Dynabeads and total RNA in 6 x SSC solutions were heated for 10 min at 75℃. Thereafter, the suspension was incubated at room temperature for 3 hr to allow binding of the U2 snRNAs to the dynabeads. The oligonucleotide-coated Dynabeads were then washed, in succession, three times with 3 x SSC, twice with 1 x SSC, and several times with 0.1 x SSC. U2 snRNA retained on the beads was eluted three times using RNase-free water [3]

U2 probe: biotin-atactacactttgatcttagccataaggcc

LC-MS/MS sample preparation and analysis

100ng U2 snRNA was digested with 1U of nuclease P1 (Sigma) in the indicated buffer in 30 uL reaction at 37℃ for 3 hours. Then 1U of Antarctic Phosphatase (NEB) was added to a final reaction volume of 100 μL with

phosphatase buffer (NEB) for an hour at 37℃ to dephosphorylate the U2 snRNA. After incubation, 100 µL samples were filtered with Millex-GV 0.22u filters. The samples were run in mobile phase buffer A (water with 0.1% Formic Acid) and 2 to 20% gradient of buffer B (Methanol with 0.1% Formic Acid). MRM transitions were measured for adenosine (268.1 to 136.1, retention time 1.03 min), N6-methyladenosine (m6A) (282.1 to 150.1, retention time 1.79 min), N6,2-O-dimethyladenosine (m6Am) (296.1 to 150.1, retention time 2.40 min). The concentrations of each compound in the samples were calculated using calibration curves constructed with standard compounds of adenosine (Abcam), N6-methyladenosine (Abcam), N6,2'-O-dimethyladenosine (Toronto Research Chemicals). Agilent Mass Hunter LC/MS Data Acquisition Version B.08.00 and Quantitative Analysis Version B.07.01 softwares are used for data collection and analysis.

Kinetics analysis

The kinetic parameters for METTL4 mediated RNA methylation were determined by incubating full-length METTL4 enzyme (300 nM) with increasing concentrations (500 nM – 10 µM) of the substrate (ATCGCTTCTCGGCCTTATGGCTAAGATCAAAGTGTAGTATCTGTTCT) at room temperature in a buffer containing 10 mM HEPES (pH 7.4 @ 25 °C), 5 mM DTT, and 100 µM S-adenosylmethionine (SAM). Aliquots were withdrawn at t = 0, 5, 10, 30, 90 and 150 min and boiled for 3 min to stop all enzymatic activity. The aliquots were further processed according to the LC-MS/MS sample preparation. The levels of the final product (m6A) formed at each time point was determined using mass spectrometry by monitoring the amount of m6A in each aliquot. The observed rate of product formation (kobs) was determined by plotting the concentration of m6A against time for each concentration of the substrate (0.5, 0.75, 1.50, 2.50, 5, 10 µM). The kobs vs substrate concentration curve was fit to the Michaelis-Menten equation for substrate inhibition kinetics using the Graphpad Prism software to obtain the final enzymatic parameters.

eCLIP-seq experiment and data processing

eCLIP-seq experiment was done by Eclipse BioInnovations Company using Sigma M2 anti-FLAG antibody and 10M cells per experiment. Western blot of immunoprecipitation was done during Flag-tag eCLIP in Kc D. Melanogaster cells. 15% of Flag-tag IP, and 1% of input were run on NuPAGE 4-12% Bis-Tris protein gels, transferred to NC membrane, probed with 1:4000 M2 anti-Flag primary antibody (Sigma, #F1804-200UG, lot # SLBT7654) and 1:10000 Mouse TrueBlot ULTRA: Anti-Mouse Ig HRP secondary antibody (Cat # 18-8817-33, Rockland Immunochemical), and imaged with C300 Imager using Azure Radiance ECL. Only the region from 50 to 125 kDa (protein size to 75kDa above) was isolated during eCLIP. Sequencing was performed as SR75 on the HiSeq 4000 platform. Raw sequencing data is processed as descripted

previously [4]. Briefly, raw fastq data was aligned to the fly reference genome dm6 together with a pool of consensus sequences for rRNA, tRNA, snRNA, snoRNA, miRNA, and LncRNA after trimming adapter and low quality reads. Mapped reads on each region was then converted to reads per million (RPM) for the correction of different sequencing depth among samples. Enrichment score was calculated by
$(RPM_{IP}-RPM_{IP\_INPUT})/(RPM_{CONTROL}-RPM_{CONTROL\_INPUT})$.

RNA-seq experiment and data processing
Total RNA was extracted with TRIzol according to the manufacturer's instructions (Invitrogen). PolyA(+) mRNA was isolated from total RNA using the NEBNext® Poly(A) mRNA Magnetic Isolation Module (NEB #E7490). The RNA-seq library preparation was carried out using the NEBNext Ultra II Directional RNA Library Prep Kit (NEB). RNA-seq was carried out on Illumina HiSeq platform with single-end 75bp read length. Raw reads were stripped of adaptor sequences and low quality bases (Q<=20) were removed using Cutadapt (https://cutadapt.readthedocs.io/en/stable/guide.html). The processed reads were aligned to fly genome (dm6) with STAR aligner (version 2.7.0f) and genes with differential alternative splicing between WT and KO cells was identified using RSEM (version 1.3.0). Significantly differentially spliced events are defined as FDR < 0.05 and fold change of exon usage > 2.

Cell proliferation assay
Cells were maintained in Drosophila medium. Cell proliferation assay was carried out by seeding 200ul of Mettl4 wild type and Knock-out cells at 60000 cells/ml in 96-well plate for 5 days. Cell number was measured every day by CyQUANT direct cell proliferation assay kit (life technologies; catalogue no: C35011).

Knock-down of mettl4 by RNAi
PCR templates for dsRNAs against Mettl4 (Amplicon ID: DRSC15026 and DRSC26716) were prepared using the MEGAscript T7 Transcription Kit (Invitrogen, AMB13345) and purified using MEGAClear (Ambion). DsRNA against the bacterial β-galactosidase gene (lacZ) was used as a control. Kc cells were dispensed into assay plates containing dsRNAs at a standard concentration for the 'bathing' method (https://fgr.hms.harvard.edu/drsc-cell-rnai). After 72 h of dsRNA incubation, CellTiter-Glo reagent (Promega) was added to each well, and luminescence was measured with a SpectraMax Paradigm Microplate Detection Platform (Molecular Probes).

References:

[1] Doxtader, K. A. *et al.*, Structural Basis for Regulation of METTL16, an S-Adenosylmethionine Homeostasis Factor. *MOL CELL* **71** 1001 (2018).

[2] Studier, F. W., Protein production by auto-induction in high density shaking cultures. *Protein Expr Purif* **41** 207 (2005).

[3] Liu, F. *et al.*, ALKBH1-Mediated tRNA Demethylation Regulates Translation. *CELL* **167** 1897 (2016).

[4] Van Nostrand, E. L., Huelga, S. C. & Yeo, G. W., Experimental and Computational Considerations in the Study of RNA-Binding Protein-RNA Interactions. *ADV EXP MED BIOL* **907** 1 (2016).
